# Evaluation of chemerin levels in the pathogenesis of psoriasis

**DOI:** 10.3389/fmed.2026.1748469

**Published:** 2026-02-18

**Authors:** Mateusz Matwiejuk, Agnieszka Kulczyńska-Przybik, Bartłomiej Łukaszuk, Hanna Myśliwiec, Piotr Myśliwiec, Adrian Chabowski, Barbara Mroczko, Iwona Flisiak

**Affiliations:** 1 Department of Dermatology and Venereology, Medical University of Bialystok, Bialystok, Poland; 2 Department of Neurodegeneration Diagnostics, Medical University of Bialystok, Bialystok, Poland; 3 Department of Physiology, Medical University of Bialystok, Bialystok, Poland; 4 1st Clinical Department of General and Endocrine Surgery, Medical University of Bialystok, Bialystok, Poland

**Keywords:** chemerin, inflammation, protein, psoriasis, skin diseases

## Abstract

Psoriasis is a complex, chronic, inflammatory condition which affects skin, nails and joints. In our study, we enrolled fifty patients with psoriasis and twenty-eight healthy individuals. Serum samples were collected both from the psoriatic patients (study group) and patients with an inguinal hernia (control group). The level of chemerin in the serum was measured by enzyme-linked immunosorbent assay. In the current research we noticed that serum chemerin concentration was significantly higher in the patients suffering from psoriasis in comparison to the controls. Importantly, we observed a positive, statistically significant correlation between the serum chemerin levels and C-reactive protein, as well as chemerin levels and platelets in the serum of patients affected by psoriasis. However, we did not observe a significant correlation between chemerin level and the Psoriasis Area and Severity Index score. To sum up, our results revealed that chemerin levels vary significantly in the serum of patients with psoriasis in contrast to the control group.

## 1 Introduction

Psoriasis is a complex, chronic, inflammatory, disease with a pathogenesis involving immune dysregulation, hyperproliferation of keratinocytes, and angiogenesis (1). Psoriasis is a skin condition with a global prevalence of approximately 2%–3% (2). Importantly, its incidence increased by 26.53% from 1990 to 2019 (3). Psoriasis can manifest at any age, with a typical onset observed in early adulthood. Literature data indicate its higher prevalence in developed countries, which likely stems from a combination of genetic predisposition, lifestyle factors (e.g., stress, diet, or smoking), and environmental triggers (e.g., infections or climate) (4, 5). Since psoriasis is a multisystemic chronic inflammatory disorder, its effects are observed beyond the skin itself. The disease is frequently linked with a higher incidence of serious comorbidities, like: cardiovascular diseases, metabolic disorders, malignancies and psoriatic arthritis (6–8). Psoriatic patients generate on average of €5,365 in healthcare costs in France, which is roughly twice as much as the control individuals without the condition (€2,682.50) (9). Psoriatic skin lesions are characterised by sharply demarcated, erythematous, scaly plaques. The areas commonly affected by psoriasis are: scalp, trunk, gluteal fold and extensor surfaces (9). While the classic symptom of psoriasis is a silvery-white scale, the actual appearance of the skin can vary significantly, it may appear as micaceous, thin or thick scale (10). Psoriasis is primarily driven by T-cells presence, specifically through the convergence, overlap, and cross-talk between the Th1 and Th17 pathways (11). Moreover, psoriasis pathogenesis is an inflammatory process involving a complex interplay of immune cells (DCs, T-cells, particularly Th17, and TRMs) and cytokines (TNF, IL-23, IL-17, and IL-22), along with the involvement of antimicrobial peptides and key signalling pathways like STAT3, which all contribute to the characteristic skin pathology (12). In addition, recent studies have identified that chemerin could be a potential key player in the etiology of the condition (13, 14). Chemerin is an adipokine, a protein secreted by adipose tissue (15). Initially, it is synthesized as a 143-amino-acid proprotein (prochemerin), which undergoes further processing, by various proteases (like elastase, cathepsin G, proteinase 3, or thrombin), in the extracellular space. This subsequent cleavage typically occurs at its C-terminus, resulting in shorter, more active forms of chemerin (e.g., chemerin-25, chemerin-27). These shorter forms are the ones that bind with higher affinity to its main receptor, ChemR23, to exert its biological effects (16). Importantly, chemerin is a protein with a dual role in inflammation, acting as both a pro- and anti-inflammatory agent (17). Chemerin acts as a pivotal molecule in the early pathogenesis of psoriasis, bridging the innate immune response (neutrophil activity) with the adaptive immune response (plasmacytoid dendritic cells recruitment). Moreoverm it serves as an activated chemoattractant, primarily manufactured by dermal fibroblasts in the psoriatic lesional skin (18). Chemerin, by promoting monocyte-endothelial cell adhesion, plays a direct and significant role in the very early, crucial steps of atherosclerotic plaque initiation and progression (19). Latest studies revealed that that circulating chemerin levels are elevated in a wide range of metabolic and inflammatory diseases, including: metabolic syndrome (20), obesity (21, 22), diabetes mellitus (21), non-small cell lung cancer (23) and cardiovascular diseases (24).

The aforementioned studies indicate chemerin’s role in the pathogenesis of psoriasis, however, its precise function and the link between its serum levels and the disease severity remain unknown.

This article aims to deepen our understanding of the role of chemerin in the development and progression of psoriasis. We evaluated the concentration of chemerin in patients with psoriasis in comparison to a control group and presented its association with PASI scores (indicators of disease severity), as well as with different biochemical and clinical parameters of the patients.

## 2 Materials and methods

50 patients (20 females and 30 males) with active plaque-type psoriasis, at a median age of 51.0, and 28 healthy controls (24 females and 4 males) at a median age of 42.0 were enrolled in the study. The severity of psoriasis was estimated using PASI (25). BMI was calculated based on self-reported weight and height using standard formulas available in the literature (26). Patients with psoriasis had not received any prior systemic treatment with methotrexate, acitretin, cyclosporine, or biologic drugs. None of the patients or controls was under dietary restriction. History of chronic diseases like hypertension, liver disease (e.g., non-alcoholic fatty liver disease, cardiovascular disease, diabetes mellitus), and results of the laboratory tests were collected from hospital records of the patients, and healthy patients were excluded from the study. Laboratory tests were performed before the treatment. All psoriatic and healthy patients signed their written informed consent before enrolment in this study. The research protocol was approved by the local university bioethical committee (no APK.002.272.2025), and followed the principles of the Helsinki Declaration. Peripheral blood samples were collected after an overnight fast and before the initiation of treatment. After centrifugation, the serum has been stored at −80 °C until it was further analyzed.

### 2.1 Chemerin analysis

Serum chemerin levels (pg/mL) were measured using the Human Chemerin Quantikine ELISA (R&D Systems) following the manufacturer’s instructions in the Department of Neurodegeneration Diagnostics. Diluted serum samples (1:100) and standards were assayed in duplicate, with a coefficient of variation (CV) < 20%. Absorbance was read using Synergy2 Biotek microplate reader at 450 nm, with wavelength correction set to 540 nm. The standard curve a four parameter logistic (4-PL) curve-fit was generated using Gen 5 software to analyzed the results. The concentrations of the samples were multiplied by dilution factor (100-fold).

### 2.2 Statistical analysis

The data in Table 1 are presented as the median and interquartile range (first and third quartiles). Categorical variables (Table 1) were presented as counts and were compared using *χ2* test with Yates continuity correction. The data presented on boxplots are expressed as median (middle horizontal bar), interquartile range (box), and whiskers (1.5 * IQR). The between group comparisons for continuous variables (boxplots and Table 1) were made with Student’s *t*-test or Wilcoxon test. The choice of the test was made based on the fulfillment of normality (assessed with Shapiro–Wilk’s test) and variance homogeneity (estimated with Levene’s test) criteria. The correlation analysis (heatmaps) was constructed based on Pearson’s correlation coefficients. The obtained *p*-values for correlations were adjusted for multiple comparisons (Benjamini–Hochberg correction). A set of selected statistically significant correlations was presented on scatterplots with a trend line (obtained from linear regression) overlaid on them. The obtained *p*-value < 0.05 was deemed to be statistically significant.

**Table 1:** Clinical and biochemical characteristics of the control group (CTRL) and psoriatic patients (PSO).

Clinical and laboratory features	CTRL	PSO
Age [years]	42.0 (37.5–47.2)	51.0 (34.2–66.0)
Weight [kg]	69.50 (63.8–79.2)	84.0 (75.5–95.8)^a^
Height [cm]	165.0 (161.5–170.2)	172.5 (164.2–176.0)^a^
BMI [kg/m^2^]	25.1 (23.5–27.9)	29.0 (23.9–31.8)^a^
CRP [mg/dL]	1.0 (1.0–2.0)	3.2 (1.5–6.9)^a^
Glucose [mg/dL]	86.5 (78.8–91.0)	85.0 (80.0–93.0)
TG [mg/dL]	73.0 (67.5–82.0)	116.0 (86.2–134.5)
AST [U/L]	17.5 (15.0–21.0)	20.0 (16.2–27.0)^a^
ALT [U/L]	15.5 (11.5–18.2)	19.0 (14.2–27.8)^a^
PASI score	—	7 patients—PASI<10 26 patients—PASI 10–20 17 patients—PASI > 20
Gender [no. female/ no. male]	24/4	20/30^a^

Data are presented as median and interquartile range. ^a^Different *vs.* PSO (*p* < 0.05); BMI, body mass index; CRP, C-reactive protein; TG, triacylglycerol; AST, aspartate transaminase; ALT, alanine transaminase.

## 3 Results

In this study were included 50 patients (20 females and 30 males) with active plaque-type psoriasis and 28 healthy patients (24 females and 4 males) were included. The median age in the control group was 42, interquartile from 37.5 to 47.2 years; the median age in the psoriatic group was 51, ranging from 34.2 to 66.0 years. The average duration of psoriasis was 16 years. In the control group, the median body mass was 69.5 kg (63.8–79.2) kg, the mean height was 165.0 (161.5–170.2) cm, and the median body mass index (BMI) was 25.1 (23.5–27.9) kg/m^2^. Most of the patients from the control group (*n* = 12) (43%) had a normal weight, 11 (39%) were overweight, and 5 (18%) suffered from obesity. In the psoriasis group, the median body mass was 84.0 (75.5–95.8) (kg), the mean height was 172.5 (164.2–176.0) (cm), the median BMI was 29.0 (23.9–31.8). Most of the patients dealing with psoriasis (*n* = 19) suffered from obesity (38%), 17 (34%) were overweight, and 14 (28%) had a normal weight. The examined group, 7 (14%) patients had a mild (psoriasis area and severity index (PASI < 10)) form of psoriasis, 26 (52%) suffered from moderate psoriasis (PASI 10–20), and 17 (34%) had a severe (PASI > 20) form of psoriasis. We observed a few gender-specific sex-specific differences. Both the control and psoriatic group body heights and body weights (but not BMIs) were greater in males than in females (*p* < 0.05). In the control group males had a slightly higher blood glucose level (medians: 100 *vs.* 86 mg/dL, *p* < 0.05). In the psoriatic group males had a slightly greater TG level (medians: 124 *vs.* 96 [mg/dL], *p* < 0.05).

In the following study, we relied on the same control and study groups as in the following published article by Matwiejuk et al. (27).

**Figure 1:**
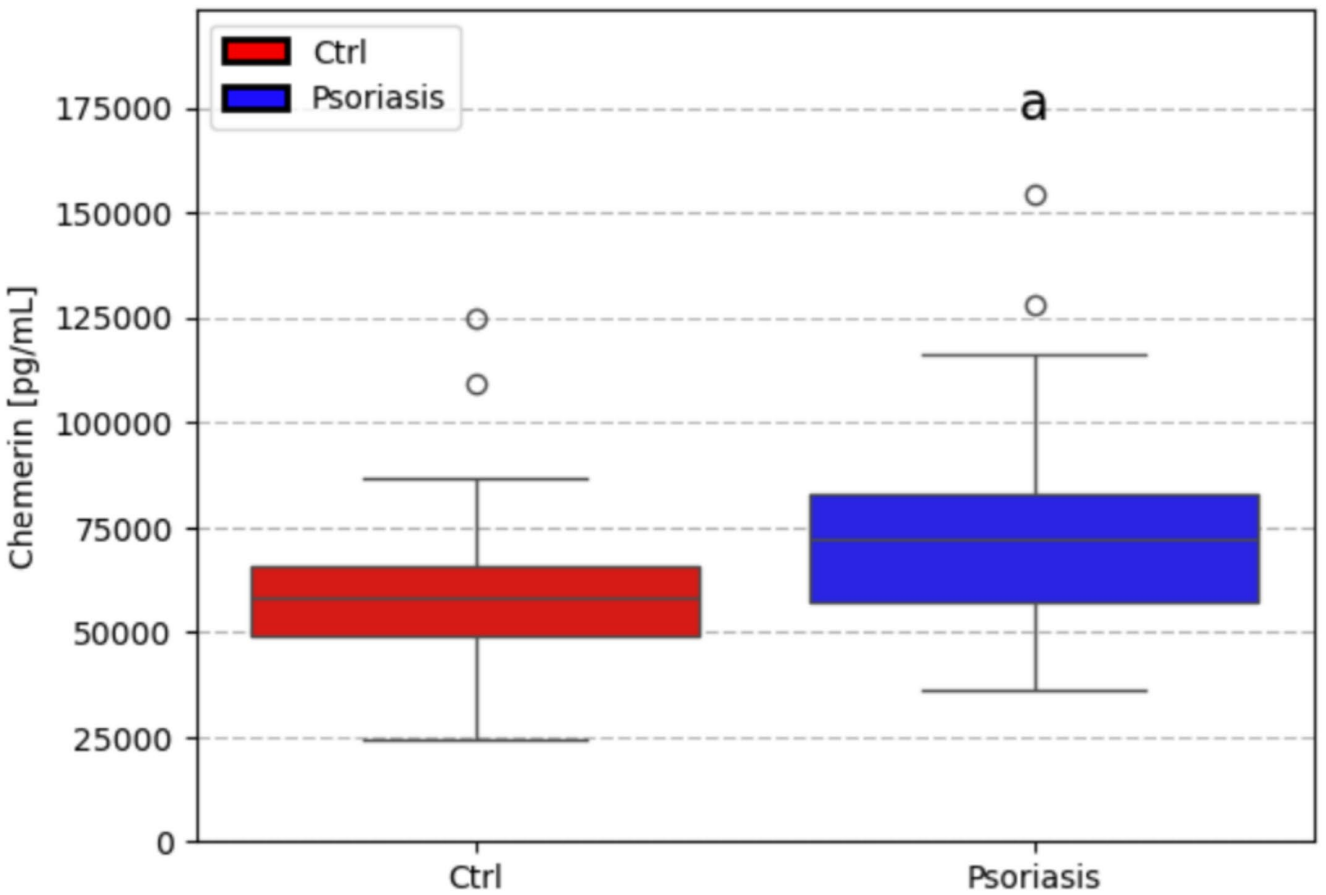
Comparison between chemerin level in healthy (CTRL) patient’s serum, and psoriatic patient’s serum [pg/ml]. a- *p* < 0.05.

**Figure 2:**
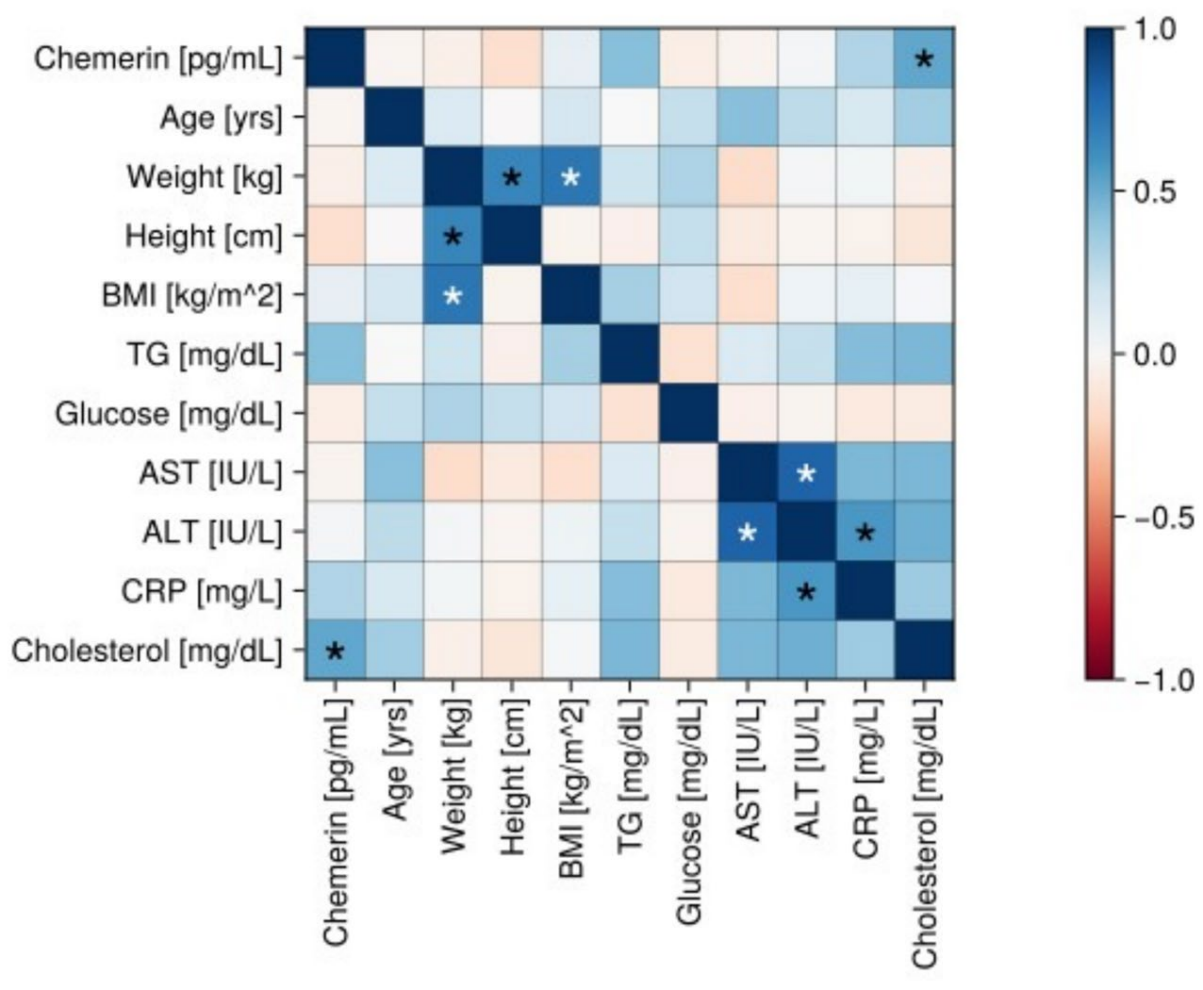
Correlation matrix (heatmap) in the control group. Pearson correlation coefficients are depicted as the shades of blue (positive correlation) or red (negative correlation). BMI, body mass index; CRP, C-reactive protein; TG, triacylglycerol; AST, aspartate transaminase; ALT, alanine transaminase.

**Figure 3:**
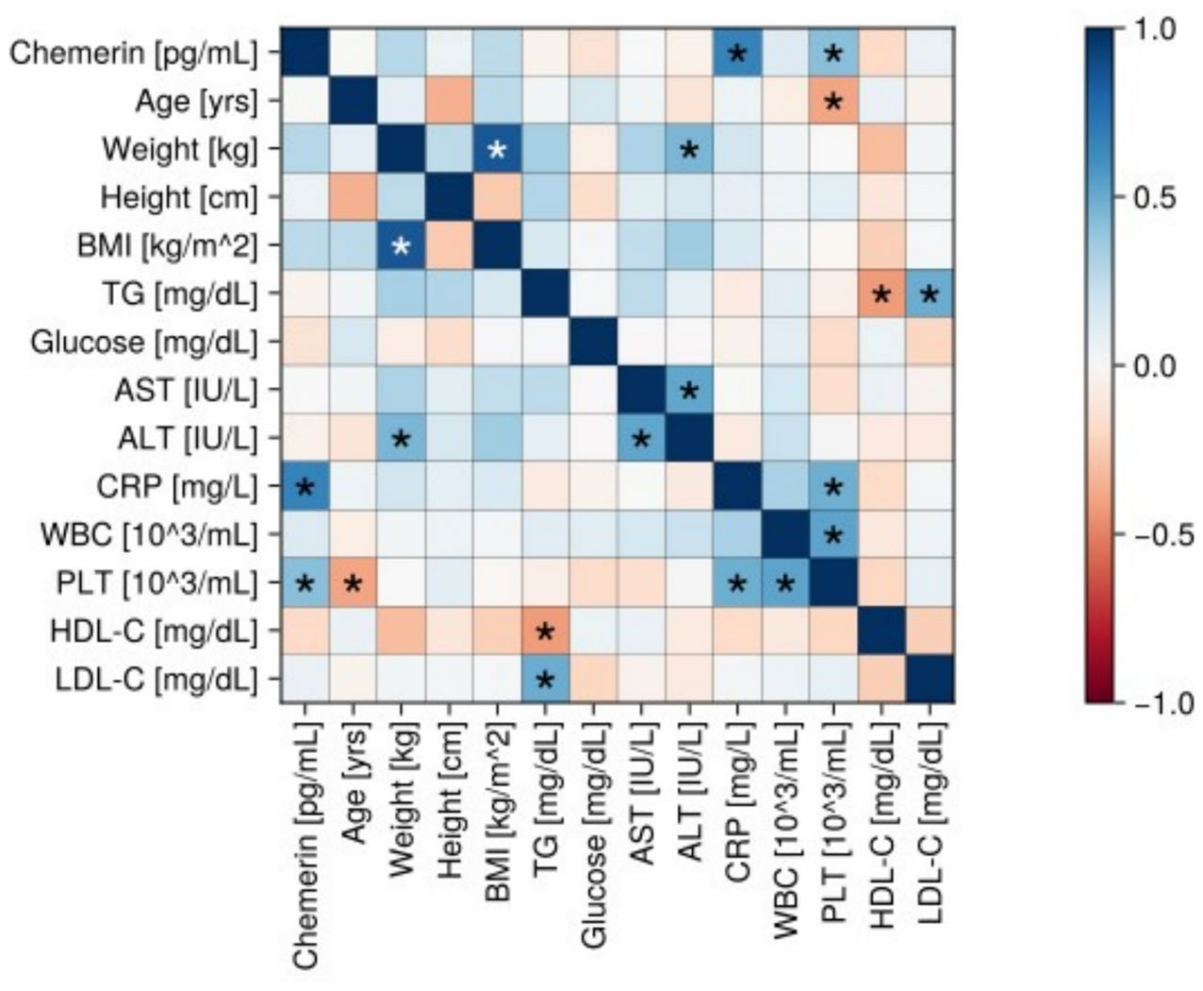
Correlation matrix (heatmap) in the psoriatic group. Pearson correlation coefficients are depicted as the shades of blue (positive correlation) or red (negative correlation). BMI, body mass index; CRP, C-reactive protein; TG, triacylglycerol; AST, aspartate transaminase; ALT, alanine transaminase; WBC, white blood count; PLT, plateles; HDL-C, high-density lipoprotein cholesterol; LDL-C, low-density lipoprotein cholesterol.

**Figure 4:**
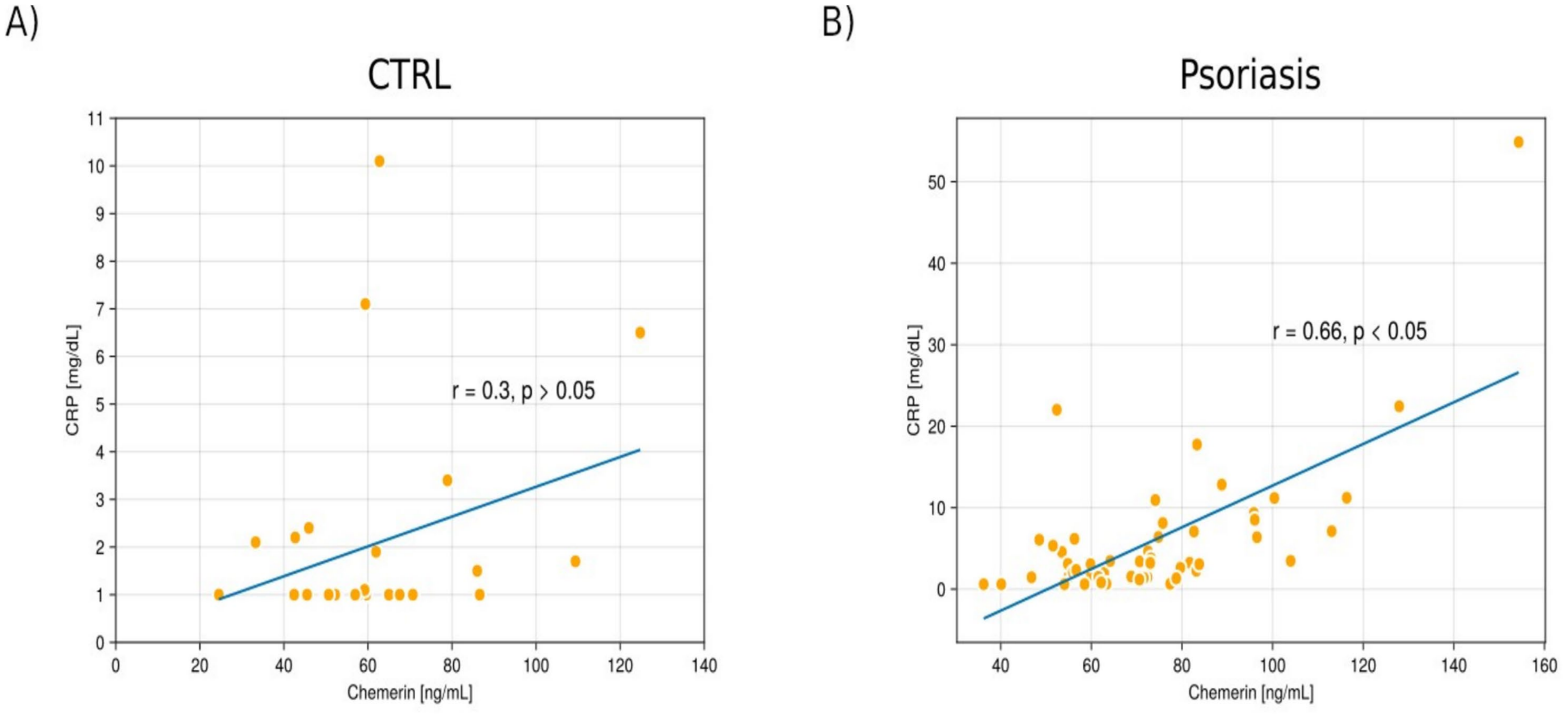
The scatterplot shows a correlation between chemerin and the level of C-reactive protein (CRP) in the serum of patients in the control group **(A)** and in patients with psoriasis **(B)**.

**Figure 5:**
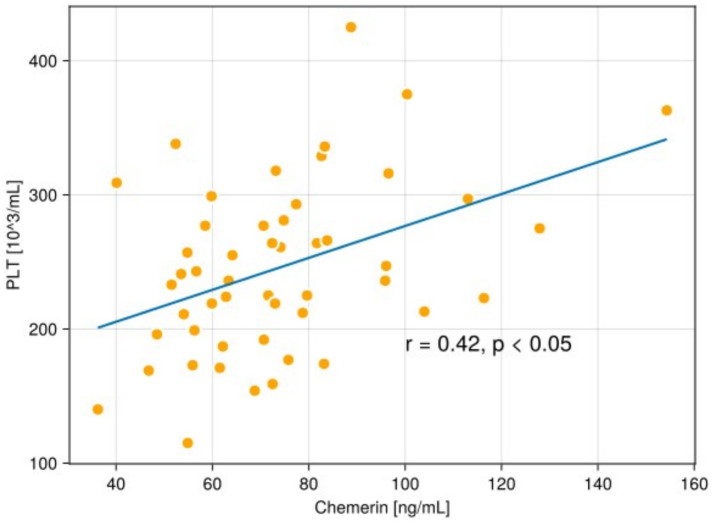
The scatterplot shows a correlation between chemerin and the level of platelet count (PLT) in the serum of patients with psoriasis.

Table 1 and Figures 1–5 summarise the main clinical features of the psoriatic group and the control group.

### 3.1 Chemerin parameter

The median concentration of chemerin found in the serum of psoriatic individuals (72001.0 pg/mL) was found to be significantly higher (*p* < 0.05) than in the serum of healthy patients (58034.3 pg/mL). Figures 2–5 present various correlations in the control and psoriatic group.

In the control group, a significant positive correlation was found between chemerin levels and cholesterol level in the serum (*r* = 0.52, *p* < 0.05) (Figure 2).

In the psoriatic group, a statistically significant positive associations between chemerin and CRP level in the serum (*r* = 0.66 *p* < 0.05), and PLT (*r* = 0.42, *p* < 0.05) were revealed (Figures 3–5).

## 4 Discussion

### 4.1 The role of chemerin

In this research, we showed that serum chemerin levels in patients with psoriasis are higher than those found in the controls. Additionally, we also assessed the link between chemerin levels and various clinical and laboratory parameters within the psoriatic patient group, which, to the best of our knowledge, has not been so principally described.

Subsequently, our research emphasised a statistically significant positive correlation between chemerin and CRP (*r* = 0.66, *p* < 0.05), and chemerin and PLT (*r* = 0.42, *p* < 0.05) in the serum of the psoriatic group. We did not find any correlation between chemerin amount in the serum level and disease severity measured by PASI.

Kong et al. (28) revealed in their study that chemerin enhanced keratinocyte proliferation, increased the production of inflammatory cytokines (IL-1beta, IL-6, TNF-*α*, IL-22, LCN2, S100A9, S100A8, and S100A7). Moreover, chemerin activated the MAPK signalling pathway, which has an overall effect that leads to an aggravation of psoriasis, through the secretion of chemokines. Interestingly, an intraperitoneal administration of a neutralising anti-chemerin antibody decreased epidermal proliferation and inflammation in an imiquimod (IMQ)-induced psoriatic mouse model (28).

Borsky et al. (29) reported significantly higher levels of chemerin found in the serum of a group of psoriatic patients (*p* < 0.05) in comparison to the serum of healthy individuals. A negative relationship was observed between chemerin levels and the PASI score. This suggests that as chemerin levels increased, the severity of psoriasis (as measured by PASI) tends to decrease (29). However, our study did not confirm these results, as we did not observe a significant correlation between chemerin concentration and PASI score.

Similarly to our study, Gisondi et al. (14) noticed that the concentration of chemerin in the serum of patients suffering from psoriasis was significantly elevated in the patients with psoriatic arthritis compared to those with psoriasis alone (195.5 ± 49.1 ng/mL *vs.* 158.1 ± 37.5 ng/mL, *p* = 0.01). Treatment with infliximab caused a significant decrease in chemerin amount and CRP levels (*p* < 0.01) (14). In line with these observations, our results also support the concept that chemerin reflects systemic inflammatory activity in psoriasis, although we did not demonstrate an association with PASI score. Taken together, their findings and our observed correlation between chemerin and CRP suggest that patients with psoriatic arthritis may experience a higher degree of systemic inflammation. Furthermore, the reduction of chemerin following anti-TNF treatment may indicate its potential utility as a biomarker of inflammatory burden in this subgroup of patients.

Consistently, Bai et al. (30) also demonstrated that mean serum chemerin levels were significantly higher in psoriatic patients compared with healthy controls. Similarly, Aksu et al. (31) reported elevated serum chemerin concentrations in psoriasis compared to controls (332 ± 73 ng/mL *vs.* 301 ± 60 ng/mL; *p* = 0.04).

Serum chemerin was positively, significantly correlated with: age, body mass index (BMI), systolic blood pressure, diastolic blood pressure, waist circumference, early diastolic peak velocity of mitral inflow/early diastolic mitral annular velocity (E/E′, which is an indicator of elevated left ventricular filling pressures/diastolic dysfunction) and epicardial fat tissue. In general, these outcomes highlighted that higher chemerin level in the serum of psoriatic patients is linked: cardiovascular risk factors, impaired diastolic function, increased epicardial fat, and endothelial dysfunction, highlighting its potential role in the cardiovascular comorbidities of psoriasis (31).

Nakajima et al. (13) showed that the circulating levels of chemerin were significantly raised in the patients with psoriasis in comparison to the individuals with chronic dermatitis and healthy control groups. Chemerin levels were observed to be lower following cyclosporine treatment administered to the psoriasis patients. However, this reduction in chemerin levels reversed after the treatment with cyclosporine was withdrawn, which furthermore points out a possible connection between the drug’s effect and chemerin levels. Moreover, the authors observed that alterations in circulating chemerin levels were associated with changes in the PASI score (13).

Tekely et al. (32) observed that serum chemerin concentration in patients with psoriasis was significantly higher than in healthy controls (*p* = 0.0003). In addition, a significant negative correlation was spotted between chemerin serum levels and high-density lipoprotein cholesterol in psoriatic patients. Furthermore, a statistically significant positive correlation was found between chemerin and triglycerides in psoriasis patients. These results showed that raised chemerin levels in psoriasis are associated with an unfavourable lipid profile, potentially contributing to the increased cardiovascular risk observed in these patients (32).

Zeid et al. (33) observed that plasma chemerin levels was significantly higher in psoriasis patients compared to controls (*p* < 0.001). Plasma chemerin amount had a significant negative correlation with the longevity of psoriasis (*r* = −0.517, *p* = 0.02), which points out that elevated plasma chemerin levels might be more prominent in the beginning of psoriasis, or that its levels might lower with the disease progression (33).

Nevein et al. (34) noticed that the serum level of chemerin was significantly higher in patients with psoriasis in comparison to the control group (*p* < 0.05). Moreover, the authors presented a significant, positive correlation between PASI and chemerin level (34).

Wang et al. (35) reported that chemerin and its receptor, chemR23, had higher expression in the serum of patients with psoriasis in comparison to healthy individuals. The ratio of Th9 cells to regulatory T cells was significantly higher in the examined group than in the healthy controls (*p* < 0.05). Interestingly, treating the CD4 + T cells with 150 ng/mL of chemerin significantly surged the levels of pro-inflammatory cytokines (IL-6, IL-9, and IL-17, *p* < 0.05). Moreover, this treatment also caused an elevated Th9/Treg ratio (*p* < 0.05), thus disrupting the immune cell balance. To clarify, the authors presented the effects of chemerin on CD4 + T cells, which were reversed by silencing of chemR23, i.e., chemerin receptor (*p* < 0.05). In summary, the presented data demonstrate that chemerin possesses regulatory influence on T cells via its receptor, chemR23, and that chemerin leads to the immune abnormalities in psoriasis pathogenesis by boosting a pro-inflammatory Th9/Treg imbalance in CD4 + T cells, mainly mediated via the chemR23 receptor (35).

### 4.2 Chemerin and CRP

Gisondi et al. (14); Borsky et al. (29); Tekely et al. (32) also found and presented a correlation between chemerin and CRP level in the serum of patients dealing with psoriasis which was similar to the current study.

Gisondi et al. (14) informed that CRP is a crucial proinflammatory protein, which belongs to the pentraxin family. Hepatocytes synthesise CRP it and CRP production is mainly controlled by cytokines like IL-6 and IL-17. In this study, the authors observed increased serum CRP levels in the patients with active plaque psoriasis. This elevation is directly connected to psoriasis-related inflammation, which is evidenced by the decrease in CRP levels following successful treatment with infliximab (anti-TNF-*α* agent). Moreover, the authors underscored that elevated an CRP level is a well-established predictor of future cardiovascular and cerebrovascular incidents in healthy individuals, patients with cardiovascular risk factors, and patients dealing with chronic immune-mediated inflammatory diseases (14). Our findings stay in line with however, prospective studies are needed to evaluate the true prognostic value of chemerin in this context.

Similarly to above mentioned study, Borsky et al. (29) confirmed that the link between CRP serum level and cardiovascular incident was correlated with its elevation in plaque psoriasis and decrease after successful treatment. As a result, CRP level could be considered as an appropriate age-dependent indicator for the early detection of cardiovascular comorbidities in psoriatic patients (29).

Tekely et al. (32) reported a significant positive correlation between the inflammatory marker CRP and chemerin. Therefore, chemerin is linked with psoriatic inflammation and could be an essential marker for controlling the psoriatic inflammatory process. This outcome points out that using chemerin serum levels as a prospective indicator to monitor the severity and development of the course of this dermatosis in patients suffering from psoriasis (32).

### 4.3 Chemerin and PLT

Interestingly, in our study we demonstrated, for the first time to our knowledge, a statistically significant positive correlation between serum chemerin levels and platelet count (*r* = 0.42, *p* < 0.05) in the examined group. This novel observation has not been previously reported in the literature and may suggest a potential link between chemerin and platelet-related pathways in the context of systemic inflammation in psoriasis. To the best of our knowledge, this outcome has not been previously reported.

Fan et al. (36) observed in their study the most common, long-standing platelet hyperaggregation in psoriatic patients, a phenomenon that could contribute to thrombus formation (36). Liu et al. (37) revealed that abnormal arachidonic acid metabolism during psoriasis may trigger PLT aggregation. Precisely, the researchers revealed that an elevated production of prostaglandins G2 and H2, especially thromboxane A2, in the platelet plasma membrane, was involved in this aggregation process (37). Ozkur et al. (38) revealed that PLT was significantly higher in the patients affected by psoriasis when compared to healthy individuals (*p* = 0.012 and *p* = 0.015, respectively). Importantly, the serum amount of PLT had a positive, statistically significant correlation with PASI scores (*r* = 0.424, *p* = 0.025). These outcomes suggest that raised PLT in patients with psoriasis highlighted the participation of PLT in the course and progression of this dermatosis and emphasises its systemic inflammatory nature (38). Kim et al. (39) demonstrated that in psoriatic patients, the PASI score had a positive, significant correlation with PLT (*r* = 0.2389, *p* = 0.0116). Additionally, this parameter was significantly higher in patients with moderate to severe psoriasis (PASI ≥ 10) in comparison to the patients with mild stage of psoriasis (PASI < 10) (39). Li et al. (40) and Pektas et al. (41) did not identify a link between PLT and the severity of psoriasis (40, 41).

Further studies are warranted to confirm this association and to explore its possible pathophysiological and clinical implications.

However, several limitations of our study should be mentioned. The patients with diagnosed and/or treated cardiovascular diseases were eliminated from our study, still, a possibility exists that some of the included patients had undiagnosed or subclinical cardiovascular conditions that could have influenced both chemerin, CRP and PLT levels. For this reason, the observed correlation might reflect an early or pre-diagnostic cardiovascular disorder, potentially serving as a prognostic indicator rather than a direct mechanistic connection. Additionally, the research cohort was relatively small (50 psoriatic patients and 28 controls), which limits the statistical power and generalizability of the findings. All blood samples were collected before the start of psoriasis-specific treatment, and it is not exactly known how this precise systemic therapy might subsequently affect the chemerin-CRP and chemerin-PLT correlations. Hence, larger and longitudinal researches are needed to confirm these outcomes and to better define the underlying mechanisms.

## 5 Conclusion

We found an increased chemerin concentration in the serum of psoriatic patients. The above may indicate a key role of this protein in the systemic pathogenesis of psoriasis. This outcome may suggests that cardiovascular and inflammatory conditions in the course of psoriasis go far beyond the clinical presence of psoriatic skin lesions, involving circulating proteins such as chemerin in the disease development. These results underline the probable of role chemerin as a biomarker and possibly a therapeutic target. In addition, we reported a statistically significant positive correlation between chemerin and CRP, as well as chemerin and PLT levels in the serum of psoriatic patients. Interestingly, chemerin, which is a protein with a dual role in inflammation, acting as both a pro and anti-inflammatory agent, in our study, we confirmed its proinflammatory role in the psoriatic pathogenesis by presenting the aforementioned correlations between chemerin and CRP, and chemerin and PLT in the serum of psoriatic patients.

Larger, prospective studies are necessary to assess its eventual anti-inflammatory role in the course of psoriasis and to confirm whether this biomarker could be used prognostically or therapeutically in the management of psoriasis.

## Data Availability

The raw data supporting the conclusions of this article will be made available by the authors, without undue reservation.
